# Native myocardial T1 and ECV with age and gender developing normal reference ranges - a 94 healthy volunteer study

**DOI:** 10.1186/1532-429X-18-S1-O42

**Published:** 2016-01-27

**Authors:** Stefania Rosmini, Heerajnarain Bulluck, Thomas A Treibel, Amna Abdel-Gadir, Anish N Bhuva, Veronica Culotta, Ahmed Merghani, Viviana Maestrini, Anna S Herrey, Peter Kellman, Charlotte Manisty, James Moon

**Affiliations:** 1 Barts Heart Centre, Cardiac Imaging, London, UK; 2 grid.83440.3b0000000121901201Department of Cardiovascular Sciences, St Georges, University of London, London, UK; 3 grid.7841.aDepartment of Cardiovascular, Respiratory, Nephrology, Anesthesiology, and Geriatric Sciences, "Sapienza" University of Rome, Rome, Italy; 4 grid.94365.3d0000000122975165National Heart, Lung, and Blood Institute, National Institutes of Health, Bethesda, MD USA

## Background

Measurement of native T1 and extracellular volume fraction (ECV) allow quantification of diffuse myocardial fibrosis. Normal references ranges are not definitively established. The concept of increasing fibrosis with age is widely held, despite a lack of supportive evidence from tissue studies - some of which say the opposite (Olivetti 1991) or point to other age changes eg myocardial lipofuschin or haemosiderin accumulation.

Whether the ECV increases with age is unclear - The MESA study found a small ECV increase with age (R^2^ 0.021, p = 0.012, Lima JACC 2013) but used measurement rather than mapping, whilst others (Ugander, Schelbert) found increases but with small populations or with significant comorbidities. Using state-of-the-art mapping, we sought to determine whether T1 and ECV increase with age - both to understand the aging biology and as a step towards developing normal reference ranges.

## Methods

94 healthy volunteers with no known cardiovascular disease underwent CMR at 1.5 T (Siemens, Avanto). Mid-ventricular short axis native and post-contrast (15 minutes post 0.1 mmol/kg Dotarem) T1 maps by MOLLI and ShMOLLI were acquired. MOLLI T1 maps [pre: 5s(3s)3s, post: 4s(1s)3s(1s)2s] with motion correction were used to generate automated ECV maps as previously described (Kellman JCMR 2012).

Manual epi-and endocardial contours were drawn using CVI42 (Calgary, Canada). Every effort was made to avoid confounders, with blood partial voluming being a particular concern. We therefore initially assessed the effect of different degrees of endo- and epicardial border erosion and showed that using values >10% conferred no added value, therefore 10% was used for all studies.

**Figure 1:**
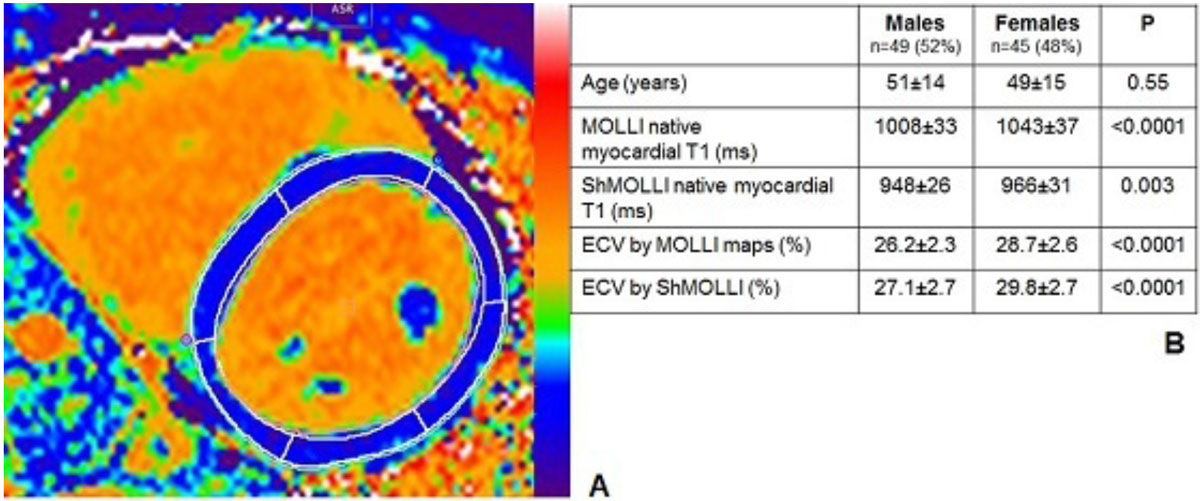
**A. Segmentation of a MOLLI ECV map : mid-short axis slice showing endocardial and epicardial border with a 10% erosion offset and divided into AHA segments**. B. Relation ship between gender and age, myocardial T1 and ECV (by MOLLI and ShMOLLI).

Contours were then exported to the post-contrast T1 maps and the MOLLI ECV map (Fig. 1A), with the mid anteroseptum used for analysis. ECV was calculated using the formula ECV=(Δ[1/T1myo]/Δ[1/T1blood])*[1-Hct]) for ShMOLLI, and using the mean segmental pixel value from the MOLLI ECV map.

## Results

Mean age was 50 ± 14, range 20-76 years, male 52%, with no gender difference in age (Figure 1B). Heart rate decreased slightly with age (R^2^ 0.075, coeff. -0.273, p = 0.008) but there was no relationship between age and other influences on blood T1 (hematocrit, iron and HDL cholesterol).

Overall mean myocardial T1 and ECV were 1025 ± 38 ms and 27.4 ± 2.8 by MOLLI and 957 ± 30 ms and 28.4 ± 3 ms by ShMOLLI, with higher values in females than males for both (Figure 1B)

Native myocardial T1 reduced slightly with age (R^2^ 0.131, p < 0.0001 by MOLLI, Figure 2A, and R^2^ 0.042, p 0.048 by ShMOLLI) - on average by 11 ms/decade by MOLLI and 8 ms/decade by ShMOLLI.

**Figure:**
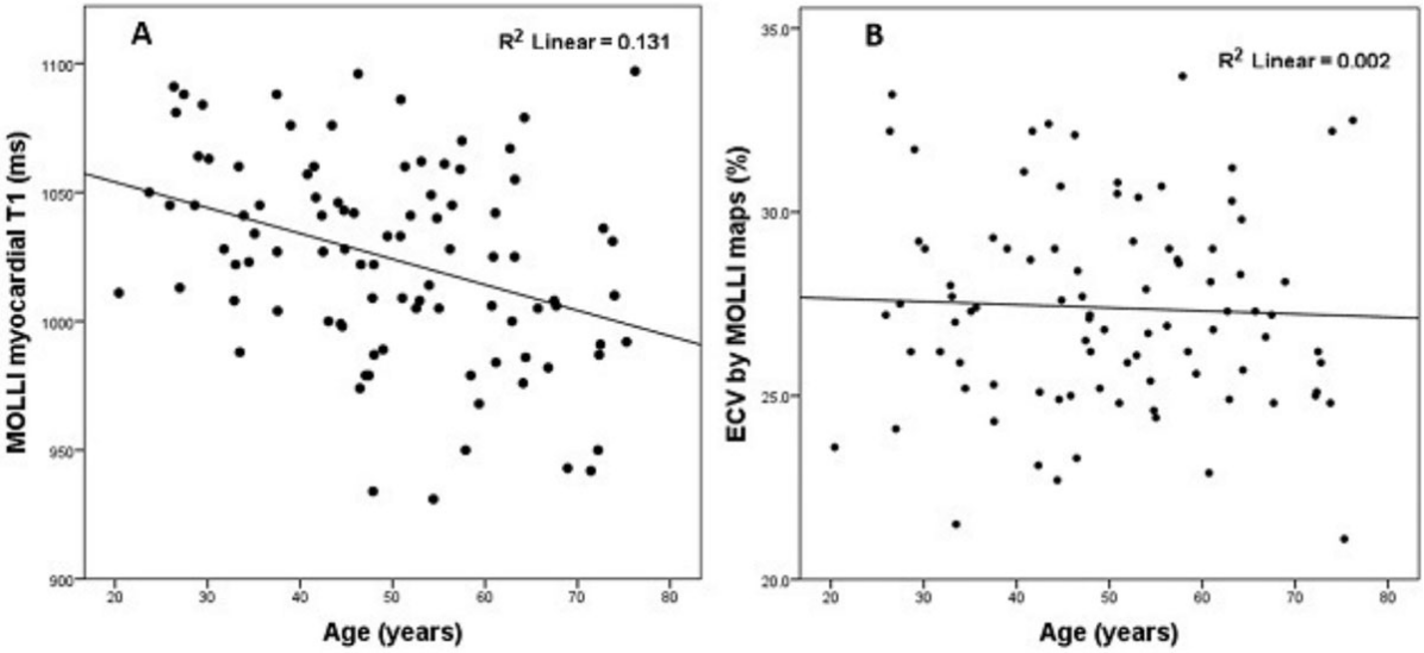
Figure 2

ECV did not change significantly with age by either MOLLI (R^2^ 0.002, p = 0.689, Figure 2B) or ShMOLLI (R^2^ 0.003, p = 0.582).

## Conclusions

Gender influences native T1 and ECV with women having a higher native T1 (+35 ms) and ECV (+0.5). Age does not influence ECV, but T1 falls slightly with age (by 11 ms/decade).

